# Models of care on the management of women with polycystic ovary syndrome: A multicentre study

**DOI:** 10.1007/s12020-026-04623-6

**Published:** 2026-05-02

**Authors:** Amynta Arshad, Eleni Armeni, Amanda Ling Jie Yee, Anisah Ali, Aspasia Manta, Seema Pandey, Agatha Chu, Sahrish Khan, Harshal Deshmukh, Erkut Attar, Fahrettin Kelestimur, Ashwin Joshi, Dimitrios G. Goulis, Meri Davitadze, Madhavi Gudipati, Samuel Sherratt-Mayhew, Isin Yesim Baylan, Kyranna Lafara, Elmira Abdullayeva, Banu Erkal, Ipek Arslan, Berfin Ece Bingul, Ege Celil Cevani, Raghavendar Palani, Alexander Wilde, Lucy Murphy, Angharad James, Punith Kempegowda

**Affiliations:** 1 Warwick Hospital, South Warwickshire NHS Trust, Warwick, UK; 2 Department of Applied Health Sciences, School of Health Sciences, College of Medicine and Health, University of Birmingham, Birmingham, UK; 3 Royal Free Hospital London NHS Foundation Trust, London, UK; 4 Queen Elizabeth Hospital Birmingham, University Hospitals Birmingham NHS Foundation Trust, Birmingham, UK; 5 Sandwell and West Birmingham NHS Foundation Trust, Birmingham, UK; 6 Seema Hospital and EVA Fertility Clinic, Uttar Pradesh Azamgarh, India; 7 Hull University Teaching Hospital NHS Trust, Hull, UK; 8 PCOS and hirsutism clinic, Yeditepe University Hospital, Istanbul, Turkey; 9 South Tyneside and Sunderland NHS Foundation Trust, Sunderland, UK; 10 1st Department of Obstetrics and Gynaecology Medical School, Aristotle University of Thessaloniki, Thessaloniki, Greece; 11 Clinic NeoLab, Tbilisi, Georgia

**Keywords:** polycystic ovary syndrome, models of care, Donabedian model, chronic care model

## Abstract

**Purpose:**

International recommendations on the treatment of polycystic ovary syndrome (PCOS) advocate comprehensive assessment, risk stratification, and patient-centred education at first presentation; however, implementation within real-world Models of Care (MoCs) remains poorly understood. This study aimed to characterise PCOS MoCs, assess alignment with international recommendations, and examine variability in investigations at first specialist consultation.

**Methods:**

We conducted a multicentre observational mixed-methods evaluation across eight secondary-care centres in five countries. Qualitative clinician interviews described MoC structures, staffing, referral pathways, and documentation practices. Quantitative analysis of retrospective clinical records from women attending their first PCOS consultation between January 2020 and December 2023 (*n* = 1,321) assessed completion of guideline-recommended biochemical investigations. MoCs were evaluated using the Donabedian framework and the Chronic Care Model (CCM), mapped across ten domains of the 2023 International PCOS Guidelines.

**Results:**

Substantial heterogeneity in PCOS MoCs was observed within and across countries. Documentation of emotional well-being screening (9.4–17.6%) and long-term cardiometabolic risk education (0.7–29.9%) was low. Donabedian analysis identified structural determinants—including funding models, multidisciplinary team availability, administrative support, and clinic organisation—as key drivers of variation in care processes. CCM mapping demonstrated limited decision support, weak care coordination, and minimal reinforcement of patient self-management. Centres with established multidisciplinary teams and structured follow-up pathways showed greater guideline alignment and more complete biochemical assessment.

**Conclusion:**

Variation in PCOS care delivery is driven by systemic structural differences rather than clinical judgment. Strengthening multidisciplinary capacity and embedding CCM-aligned processes are essential to improve guideline adherence and support equitable, person-centred PCOS care.

**Supplementary Information:**

The online version contains supplementary material available at 10.1007/s12020-026-04623-6.

## Background

Polycystic Ovary Syndrome (PCOS) is a common multisystem condition with reproductive, metabolic and psychological implications, requiring coordinated multidisciplinary care [1]. Despite its high prevalence, women with PCOS frequently report dissatisfaction with care, driven by fragmented pathways, inconsistent diagnostic practices, and variable clinician expertise [2]. These inconsistencies contribute to diagnostic uncertainty, emotional distress, and suboptimal engagement with long-term management. Structured, evidence-based Models of Care (MoCs) offer a pathway to improve the quality, consistency, and efficiency of PCOS services by supporting evidence-based assessment, standardised management and patient-centred education [3, 4]. Although international guidelines emphasise the need for such models [5, 6], real-world data on their organisation, delivery and adherence to recommendations remain scarce [7]. In the absence of standardised pathways, many women experience incomplete risk assessment, delayed diagnosis and inadequate long-term counselling, with consequences for quality of life (QoL) and healthcare utilisation [8].

The need for this work has been independently underscored by national priority-setting initiatives, including the APPG for PCOS Inquiry (2023), which called for urgent improvement in diagnostic pathways, multidisciplinary access, and equitable service provision [9]. Similarly, the James Lind Alliance PCOS Priority Setting Partnership (2021) identified major evidence gaps in long-term care models and patient-centred outcomes, further emphasising the importance and timeliness of our study [10].

Surveys of endocrinologists and gynaecologists have demonstrated wide variation in diagnostic approaches [11–13], but no studies have systematically described how MoCs are structured, or how closely they align with guideline-based PCOS care.

To address this gap, we undertook a multicentre evaluation to [1] characterise existing PCOS MoCs [2], assess their alignment with international recommendations, and [3] examine variability in the investigations completed during the first consultation for PCOS in both international and UK settings.

## Methods

This multicentre observational mixed-methods study was conducted as part of a broader service evaluation for PCOS (REC reference: 22/PR/1178) from January 2024 to February 2025. Centres that provided secondary level care to women with PCOS were invited to participate in this study. Centres were recruited through an open online call disseminated via national and international networks in endocrinology and reproductive medicine. Participation was voluntary. Centres were eligible if they: (a) Routinely managed adult women with PCOS: (b) Used structured or standardised clinical documentation (e.g., electronic templates or consistent departmental records); (c) All eligible centres that responded were included without geographic restriction. Each centre obtained local clinical governance approval prior to participation.

### Participants

All women aged ≥ 18 years who attended their first clinical consultation for suspected or confirmed PCOS in participating centres from January 2020 to December 2023 were included in the study. Only the first consultation was selected because it typically includes the most comprehensive diagnostic work-up and because, in several participating countries (e.g., India, Turkey, Greece), follow-up is inconsistent due to private-sector costs. In the UK, prolonged NHS waiting times similarly position the first appointment as the main opportunity for guideline-based assessment [5, 14, 15]. Prior literature confirms the first consultation’s central role in PCOS diagnosis, education, and pathway formation [15, 16].

## Data collection and variables

Data were obtained from two complementary sources: centre-level interviews and patient-level clinical records.

Centre-level data were collected through semi-structured online interviews with senior clinicians from each participating site. The interview guide covered service establishment, funding model, staffing, multidisciplinary availability, referral pathways, documentation systems, and routine assessment requirements. Interviews were summarised using a descriptive qualitative approach, focusing on explicit content rather than interpretive thematic analysis. Findings were presented descriptively and used to characterise the structural features of each Model of Care and to contextualise observed differences in documentation and investigation practices.

Patient-level data were extracted retrospectively from clinical records. Extracted variables included demographic characteristics (age and ethnicity), medical and reproductive history, documented diagnostic criteria, and evidence of dermatological, cardiometabolic, reproductive, emotional, and lifestyle assessments. We also recorded whether centres documented patient satisfaction processes or healthcare practitioner evaluations as part of routine quality monitoring. Biochemical and hormonal investigations performed during the first consultation were captured in detail, including androgen profile, gonadotropins, reproductive hormones, endocrine differentials, and metabolic parameters. Missing data were handled using listwise deletion, as the low proportion of missingness was unlikely to compromise statistical power or introduce systematic bias.

## Assessment of models of care

Each participating centre’s MoC was evaluated against the 10 core components derived from the 2023 version of international PCOS guidelines. These components included documentation of dermatological assessment, cardiometabolic risk evaluation, emotional well-being screening, long-term risk education, lifestyle management, reproductive screening, and adherence to the Rotterdam diagnostic criteria [5]. We also assessed whether centres incorporated multidisciplinary involvement, collected patient satisfaction data, or conducted healthcare practitioner evaluations. For each guideline-based parameter, we first recorded whether the centre formally required its assessment within routine practice. We then reviewed patient records from the first consultation to determine whether there was documented evidence that the assessment had been completed. This approach enabled comparison between the intended design of each MoC and its actual implementation in clinical practice. Documentation of assessments and investigations was evaluated as recorded in the clinical notes. As such, all outcomes reflect documentation practices rather than direct measures of care delivery.

## Assessment of biochemical investigation completion

Completion of biochemical investigations was assessed by comparing the tests performed at each woman’s first consultation, with the investigations recommended in the international PCOS guidelines [5, 17]. For each centre, we documented whether key hormonal, metabolic, and endocrine differential tests were obtained, including: Total and free Testosterone or Free Androgen Index (FAI), Androstenedione, Dehydroepiandrosterone Sulphate (DHEAS), Luteinizing Hormone (LH), Follicle-Stimulating Hormone (FSH), Sex Hormone-Binding Globulin (SHBG), Serum Progesterone, Thyroid-Stimulating Hormone (TSH), Prolactin, 17-Hydroxyprogesterone (17-OHP), Cortisol (or Dexamethasone Suppression Test)), Fasting Plasma Glucose (FPG) (or Glycated Haemoglobin (HbA1c), Total Cholesterol, Low-Density Lipoprotein (LDL), High-Density Lipoprotein (HDL), Triglycerides.

Completion rates were calculated as the proportion of recommended investigations performed during the first consultation. Completion refers to full adherence to the complete panel recommended by international guidelines, acknowledging that individualised testing may differ in clinical practice. This enabled comparison across centres and between UK and international settings, highlighting variations in the extent to which guideline-recommended biochemical evaluation was achieved.

## Conceptual framework

Interpretation of the findings was informed by the Donabedian model of healthcare quality and the Chronic Care Model (CCM). The former was applied to contextualise observed variations in PCOS care according to the domains of structure (clinic organisation and resources), process (delivery of diagnostic and management activities), and outcomes (documentation and adherence as proxies for care quality) [18]. In addition, the CCM was used to situate PCOS as a chronic multisystem condition requiring coordinated, multidisciplinary management [19, 20]. The CCM components—delivery system design, decision support, clinical information systems, self-management support, and community linkages—provided an interpretive lens to understand how system design and care processes shape service performance.

### Statistical analysis

Categorical and continuous data were processed using the Statistical Package for Social Sciences (SPSS), version 29.0. Categorical variables were expressed as percentages (%), while continuous variables were summarised as median and interquartile range (IQR). Given the large sample size and the relatively low proportion of missing data, we used listwise deletion, as the resulting loss of observations was unlikely to compromise statistical power or introduce substantial bias.

We outlined each MoC’s features, including the care setting’s structure, the staff’s clinical specialisation, the presence of a multidisciplinary team, whether the centre was affiliated with the public or private healthcare sector, and the age of participants.

To determine how closely each MoC aligns with the international PCOS recommendations [5, 6], we assessed the proportion of people with PCOS where the following parameters were evaluated: dermatological assessment, cardiometabolic risk, lifestyle management, reproductive screening, emotional wellbeing, long-term risk education, diagnosis based on the Rotterdam criteria, the presence or absence of a multidisciplinary approach, patient satisfaction, and evaluation by healthcare practitioners. For each parameter, we first identified whether the MoC formally required its evaluation, as outlined in the international recommendations [5, 6]. We then retrospectively reviewed patient records from the first consultation to determine whether there was documented evidence of this assessment. The proportion of women with documented evidence was calculated for each parameter, and comparisons were performed between international centres and, separately, across UK centres. This method allowed us to capture both the intended design of MoCs and their actual implementation in routine practice. Comparisons between the characteristics of MoCs were conducted using Analysis of Variance (ANOVA) for continuous measures or Chi-square (X^2^) for categorical variables.

To explore variability in the frequency of investigations conducted during the first consultation for PCOS, we assessed the proportion of hormonal and biochemical investigations conducted using a heatmap for all participating centres. The completion rate for biochemical investigations refers to the proportion of investigations performed during the first consultation relative to the total number of recommended investigations in the international recommendations [5, 6]. Comparisons between completion rates between centres in the UK and internationally were conducted using Chi-Square (X^2^). Statistical significance set at a p-value < 0.05 (two-tailed).

### Ethics approval and consent to participate

This study was conducted as part of a broader service evaluation for PCOS (REC reference: 22/PR/1178) and adhered to the ethical principles outlined in the Declaration of Helsinki. Local approval was obtained from each participating centre’s clinical governance or ethics committee prior to data collection. Given the retrospective design and anonymised data extraction, formal written informed consent from individual patients was waived.

## Results

### Characteristics of PCOS models of care (MoCs)

Detailed structural characteristics of the participating centres, including funding model, staffing, documentation systems, referral pathways, and quality monitoring processes, are presented in Table S1.

The participating international centres demonstrated substantial variation in funding structures, staffing, and service provision. The centre in Istanbul, Turkey (established 2019) operated as a privately funded service staffed by a reproductive endocrinologist and gynaecologist, with ad hoc input from a dermatologist and dietitian. The Thessaloniki centre in Greece (established 1982) was publicly funded and staffed by a gynaecologist, endocrinologist, and dietitian. In Tbilisi, Georgia (established 2021), PCOS care was delivered within a privately funded service led primarily by an endocrinologist. The centre in Uttar Pradesh, India (established 2013) was also privately funded and staffed by an endocrinologist, gynaecologist, general physician, and dietitian, predominantly serving women presenting with fertility-related PCOS concern.

All participating UK centres were publicly funded and operated within hospital-based services. The Birmingham centre (established 2019) functioned as an endocrinologist- and nurse-led clinic, with referrals to dermatology, gynaecology, and weight management services as required. The Hull and Sunderland centres (both established 2009) were similarly endocrinologist- and nurse-led, although Sunderland additionally included a gynaecologist within its team. The London centre (established 2000) provided PCOS care within a general endocrinology service, where initial anthropometric assessments were conducted by nursing staff prior to clinical review.

### Alignment of MoCs with international recommendations for PCOS

We evaluated 1321 women across eight centres (median age, 27 years; IQR, 24–32; 48.8% White, 48.2% Asian).

Across international centres **(**Table 1**)**, documentation of key guideline-recommended assessments was generally high. Dermatology screening was recorded for all women in Turkey and Greece and for nearly all in Georgia (96.3%), whereas the proportion was lower in the UK (80.2%). Cardiometabolic risk assessment and lifestyle management advice was documented consistently in Turkey, Greece, and Georgia (100%), with reduced completion in India (85.5% and 83.3%, respectively) and in the UK (63.2% and 63.6%). Reproductive screening showed a similar pattern, with universal documentation in Turkey and Greece, high rates in Georgia (96.3%), and lower rates in India (67.3%) and the UK (64.2%). In contrast, emotional well-being assessment was infrequently recorded, with low documentation in the UK (17.6%) and India (9.4%), and only ad hoc, inconsistently recorded evaluation in Turkey, Greece, and Georgia. Long-term risk education was more commonly documented in Turkey, Greece, and Georgia, but remained rare in the UK (17.8%) and India (0.7%). Diagnostic adherence to the Rotterdam criteria was high across the international centres (99.6%–100%), but notably lower in the UK (54%). Multidisciplinary team involvement was documented only in Turkey and Greece, while patient satisfaction assessments were recorded in Turkey, Greece, and Georgia, and healthcare practitioner evaluations were noted in Georgia and India.

**Table 1:** Comparison of care model characteristics across countries based on international recommendations

Characteristics	Overall (*N* = 1313)	UK (*N* = 506)	Turkey (*N* = 241)	Greece (*N* = 92)	Georgia (*N* = 27)	India (*N* = 447)	*p*-value (difference between countries)
Age, median (IQR)	27.0 (24.0–32.0)	30.0 (26.0–35.0)	26.0 (23.0–31.0)	23.0 (20.0–27.0)	26.0 (21.0–33.0)	26.5 (24.0–30.0)	< 0.001
**Parameters assessed**,** n (%)**							
Dermatology	1141 (86.9)	406 (80.2)	241 (100)	92 (100)	26 (96.3)	376 (84.1)	< 0.001
Cardiometabolic	1062 (80.9)	320 (63.2)	241 (100)	92 (100)	27 (100)	382 (85.5)	< 0.001
Lifestyle management	1053 (80.2)	322 (63.6)	241 (100)	92 (100)	27 (100)	371 (83.0)	< 0.001
Reproductive screening	985 (75.0)	325 (64.2)	241 (100)	92 (100)	26 (96.3)	301 (67.3)	< 0.001
Emotional wellbeing	491 (37.4)	89 (17.6)	241 (100) ^**a**^	92 (100) ^**a**^	27 (100) ^**a**^	42 (9.4)	< 0.001
Long term risk education	453 (34.5)	90 (17.8)	241 (100) ^b^	92 (100) ^b^	27 (100) ^b^	3 (0.7)	< 0.001
Diagnosis per Rotterdam criteria, n (%)	1078 (82.1)	273 (54.0)	241 (100)	92 (100)	27 (100)	445 (99.6)	< 0.001
Multidisciplinary setting		Variable	Yes	Yes	No	No	-
Patient satisfaction		Variable	Yes	Yes	Yes	No	-
Healthcare practitioners’ evaluation		Variable	no	no	yes	yes	-

IQR = interquartile range

^a^Assessment of emotional well-being was done ad hoc but was not documented. Hence, we were unable to quantify

^b^Long-term education was provided ad hoc but was not documented. Hence, we were unable to quantify

Across the four UK centres (Table 2), documentation practices varied substantially. Birmingham and Hull demonstrated the highest proportions of recorded dermatology assessments (90.2% and 86.3%, respectively) and cardiometabolic evaluations (76.6% and 100%), whereas Sunderland (37.3%, 3.9%) and London (76.0%, 24.8%) reported notably lower rates. Lifestyle advice was most frequently documented in Hull (92.5%), followed by London (56.0%) and Sunderland (52.9%), with Birmingham recording the lowest proportion (48.6%). Reproductive screening was also most consistently captured in Hull (89.0%) and least in Birmingham (49.5%), with Sunderland (62.7%) and London (57.6%) showing intermediate levels. Emotional well-being assessment and long-term risk education were infrequently documented across all sites; Birmingham reported the highest proportions (26.1% and 29.9%), while Hull reported the lowest (4.1% and 8.2%). Adherence to Rotterdam diagnostic criteria was greatest in Hull (97.3%) and lowest in Sunderland (33.3%). Notably, healthcare practitioner evaluations were recorded only in Birmingham and Sunderland, and none of the centres documented patient satisfaction measures.

**Table 2:** Comparison of the characteristics of models of care across UK centres, based on international recommendations

Characteristics	UK, Overall (*N* = 506)	Birmingham (*N* = 184)	Hull (*N* = 146)	Sunderland (*N* = 51)	London (*N* = 125)	*p*-value (difference between centres)
Age, median (IQR)	30.0 (26.0–35.0)	31.0 (28.0–36.0)	30.0 (26.0–35.0)	28.0 (25.0–33.0)	30.0 (25.0–34.0)	0.006
**Parameters assessed**,** n (%)**						
Dermatology	406 (80.2)	166 (90.2)	126 (86.3)	19 (37.3)	95 (76.0)	< 0.001
Cardiometabolic	320 (63.2)	141 (76.6)	146 (100)	2 (3.9)	31 (24.8)	< 0.001
Lifestyle management	322 (63.6)	90 (48.9)	135 (92.5)	27 (52.9)	70 (56.0)	< 0.001
Reproductive screening	325 (64.2)	91 (49.5)	130 (89.0)	32 (62.7)	72 (57.6)	< 0.001
Emotional wellbeing	89 (17.6)	48 (26.1)	6 (4.1)	5 (9.8)	30 (24.0)	< 0.001
Long term risk education	90 (17.8)	55 (29.9)	12 (8.2)	8 (15.7)	15 (12.0)	< 0.001
Diagnosis based on Rotterdam criteria, n (%)	273 (54.0)	64 (34.8)	142 (97.3)	17 (33.3)	50 (40.0)	< 0.001
Multidisciplinary setting	Variable	yes	no	yes	no	-
Patient satisfaction	Variable	no	no	no	no	-
Healthcare practitioners’ evaluation	Variable	yes	no	yes	no	-

IQR = Interquartile range

### Variability in the frequency of investigations conducted during the first consultation for PCOS internationally and in the UK

**Fig. 1:**
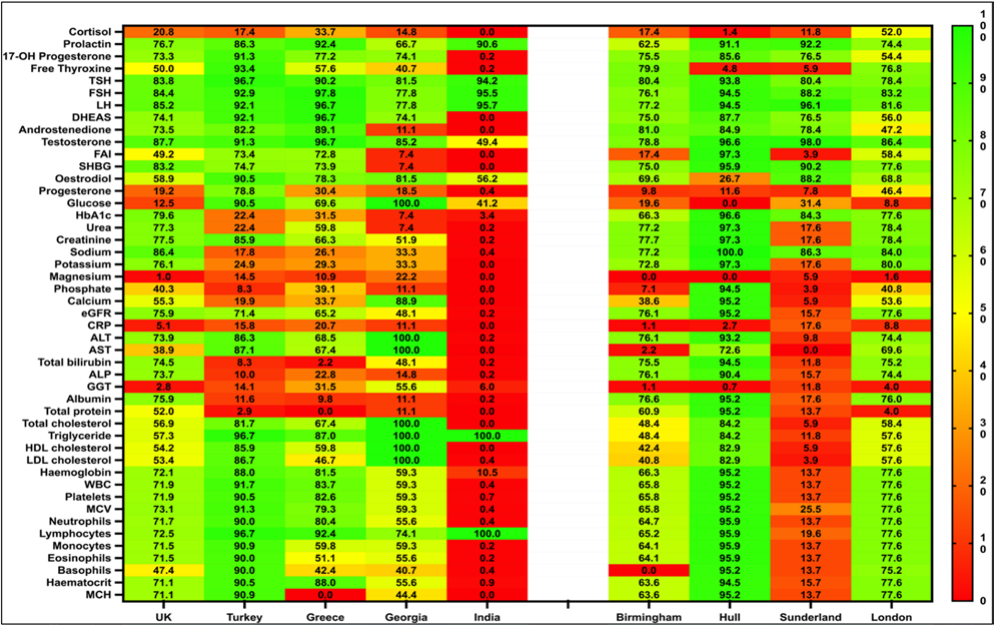
The frequency of investigations conducted for women with PCOS during their first consultation

Completion rates for recommended biochemical investigations at the first consultation varied markedly across regions. Internationally **(**Table 3**)**, the highest documentation rates were observed in Georgia and Turkey (88.2%), followed by Greece (82.4%), the UK overall (70.6%), and India (58.8%). Within the UK **(**Table 4**)**, Hull (88.2%) and Sunderland (82.4%) demonstrated the most comprehensive documentation, whereas Birmingham (70.6%) and London (58.8%) reported lower completion rates. The distribution of individual investigation completion across centres is shown in Fig. 1, highlighting substantial between-centre variability.

**Table 3:** Comparison of investigation completion rates across countries based on international recommendations

	Overall (*N* = 1313)	United Kingdom (*N* = 506)	Greece (*N* = 92)	Georgia (*N* = 27)	Turkey (*N* = 241)	India (*N* = 447)	p-value (difference between countries)
Completion rate, median (IQR) [%]	76.47 (58.82–88.23)	70.58 (58.82–82.35)	82.35 (76.47–86.76)	88.23 (88.23–88.23)	88.23 (82.35–94.11)	58.82 (52.94–76.47)	< 0.001

IQR = interquartile range

**Table 4:** Comparison of investigation completion rates across UK centres based on international recommendations

	United Kingdom, Overall (*N* = 506)	Birmingham (*N* = 184)	London (*N* = 125)	Sunderland (*N* = 51)	Hull (*N* = 146)	p-value (difference between hospitals)
Completion rate, median (IQR) [%]	70.58 (58.82–82.35)	70.58 (52.94–76.47)	58.82 (52.94–58.82)	82.35 (70.59–82.35)	88.23 (82.35–88.23)	< 0.001

IQR = interquartile range

## Discussion

This evaluation demonstrates marked heterogeneity in PCOS MoCs across both international and UK centres. Applying the Donabedian model [18] allowed us to evaluate PCOS MoCs by examining how structural attributes shaped processes of care and outcomes. Structural differences were the main drivers of heterogeneity: publicly funded centres (Greece, UK) showed more standardised referral pathways, whereas privately funded or mixed systems (India, Georgia) faced accessibility and affordability barriers, resulting in poorer follow-up [2, 21]. These findings illustrate Donabedian’s principle that “structure determines the capacity to deliver quality” [18]. Comparable evidence from Taiwan confirmed that perceived service quality depends more on staff professionalism and assurance than on physical resources [22], reinforcing that competence, communication, and coordination underpin high-quality care. Building on this diagnostic lens, the CCM provides a framework for improving care [19, 23]. The absence of institutional integration limited coordinated, long-term management in most centres- an essential CCM element [20]. Only the centre from Turkey demonstrated multidisciplinary collaboration linking endocrinology, gynaecology, dermatology, and dietetics. Elsewhere, single-speciality structures reflected a reproductive rather than chronic-care orientation [11, 12, 17]. These findings should be interpreted within the limitations of documentation-based assessments, which act as proxy indicators of care processes but may underestimate activities performed but not recorded. Process indicators, viewed through Donabedian’s model, showed variable documentation of cardiometabolic screening, lifestyle counselling, and emotional health assessment. In Donabedian terms, these gaps signal weaknesses in process quality and continuity; through the CCM lens, they reveal limited self-management support, decision-support systems, and proactive follow-up [19, 23]. Emotional well-being, central to patient-centred chronic care, was rarely assessed [24–26]. Outcome measurement was also inconsistent. Patient satisfaction and feedback were recorded only in some centres, leaving the quality-improvement loop incomplete. According to Donabedian, outcome data are essential for accountability, while from a CCM perspective, the lack of digital registries and feedback systems constrains learning and benchmarking [18–20, 27, 28]. Together, these frameworks reveal that structural fragmentation leads to process inconsistency and suboptimal outcomes, while centres with multidisciplinary teamwork and feedback systems show higher maturity across both models. Adopting both frameworks provides a roadmap for reform (Supplementary Fig. 1):

- Structure: integrate multidisciplinary teamwork and leadership [21];
- Process: standardise emotional-health and lifestyle management [29, 30];
- Outcome: embed PROMs, registries, and satisfaction surveys [27, 28].
- Viewing PCOS as a lifelong condition demands such combined quality-improvement and chronic-care principles to achieve equitable, person-centred services [17, 20, 23].

### Strengths and limitations

The study’s main strengths lie in its standardised comparison across participating centres, its evaluation of care over an extended period, and its use of mixed-methods analysis to identify gaps in service provision. While the number of participating centres, including those from the United Kingdom, may not reflect the full national spectrum of practice, the multicentre participation nonetheless offers valuable preliminary insight into real-world variation and heterogeneity in PCOS management across different healthcare settings. However, documentation bias is also possible, since certain aspects of management, such as emotional well-being assessment or lifestyle advice, may have been performed informally but not consistently recorded, leading to an underestimation of adherence. The retrospective design further limits causal interpretation and does not fully capture contextual factors influencing clinical decisions. In addition, the absence of patient-reported outcomes, such as satisfaction, quality of life, and lived experience, represents an important gap that future evaluations should address, in line with international guidance on patient-centred care.

## Conclusions

Our findings highlight heterogeneity in service delivery for PCOS. Interpreting the results through the Donabedian and Chronic Care Models highlights that variability in PCOS care arises from systemic rather than clinical factors, underscoring the need for structured, multidisciplinary, and guideline-aligned service models.

## Supplementary Information

Below is the link to the electronic supplementary material.

Supplementary Material 1

Supplementary Material 2

## Data Availability

Some or all datasets generated during and/or analysed during the current study are not publicly available but are available from the corresponding author upon reasonable request.
