# Supplementary material for: Models of care on the management of women with polycystic ovary syndrome: A multicentre study
Source: Endocrine. 2026 May 2;91(1):164. doi: 10.1007/s12020-026-04623-6 (PMC13135521; doi:10.1007/s12020-026-04623-6)

**Supplementary Figure 1.** Applying the principles of the Donabedian and Chronic Care Model framework can improve fragmented PCOS care.


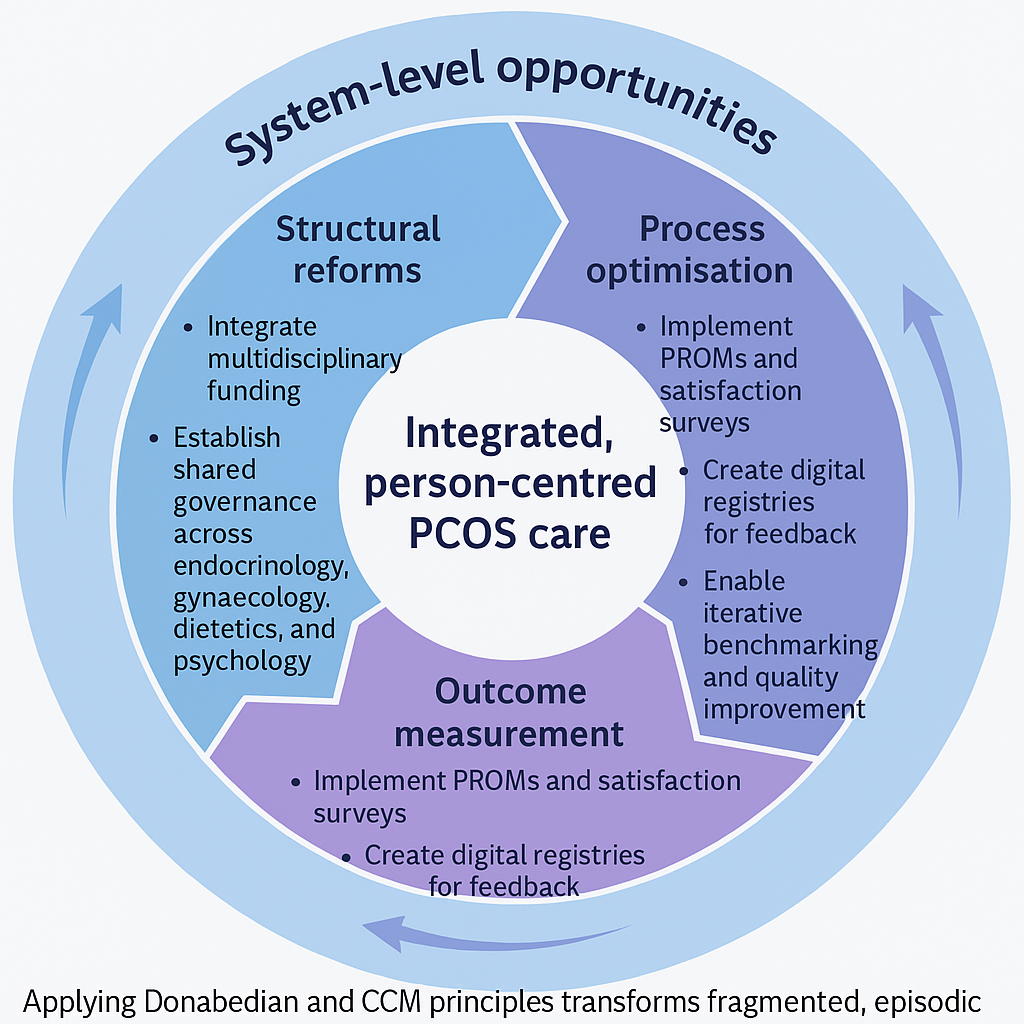

Supplement: Supplementary file 1 — Supplementary Material 1 [file 12020_2026_4623_MOESM1_ESM.docx]
