# Supplementary material for: Models of care on the management of women with polycystic ovary syndrome: A multicentre study
Source: Endocrine. 2026 May 2;91(1):164. doi: 10.1007/s12020-026-04623-6 (PMC13135521; doi:10.1007/s12020-026-04623-6)
Supplement: Supplementary file 2 — Supplementary Material 2 [file 12020_2026_4623_MOESM2_ESM.docx]

***Supplementary Table S1.*** Structural characteristics of participating PCOS centres summarised across five domains: (1) funding and governance; (2) staffing and leadership; (3) documentation systems; (4) referral pathways and integration; and (5) quality-monitoring processes. Derived from semi-structured interviews with senior clinicians and centre-level documentation summaries.*centres*.

| **Domain** | **Turkey (Istanbul)** | **Greece (Thessaloniki)** | **Georgia (Tbilisi)** | **India (Uttar Pradesh)** | **UK – Birmingham** | **UK – Hull** | **UK – Sunderland** | **UK – London** |
| --- | --- | --- | --- | --- | --- | --- | --- | --- |
| **1. Funding & Governance** | Private service; established 2019 | Public hospital; established 1982 | Private centre; established 2021 | Private clinic; established 2013 | NHS hospital; established 2019 | NHS hospital; established 2009 | NHS hospital; established 2009 | NHS hospital; established 2000 |
| **2. Staffing & Clinical Leadership** | Led by reproductive endocrinologist + gynaecologist; ad hoc derm & dietetics | Multidisciplinary: gynaecologist, endocrinologist, dietitian | Led by endocrinologist; limited MDT | Endocrinologist + gynaecologist + general physician + dietitian | Endocrinologist-led; nurse-based assessments; referrals as needed | Endocrinologist-led; nurse specialist involvement | Endocrinologist + gynaecologist; nurse involvement | General endocrinology service; nurse-led anthropometry |
| **3. Documentation & Information Systems** | Private EMR; structured templates inconsistently used | Public EMR; structured documentation | Private EMR; uniform templates | Mixed (paper + EMR); variable standardisation | NHS EPR with consistent templates | NHS EPR with structured records | NHS EPR; dermatology/gynae external notes | NHS EPR; general endocrine templates used |
| **4. Referral Pathways & Integration** | External dermatology/dietetics referrals; no formal MDT | Strong MDT integration; established pathways | Limited integration; referrals depend on affordability | Dermatology, gynae, dietetics available but cost-dependent | Dermatology, gynae, weight services via NHS referral | Good endocrine–gynae–weight pathways | Endocrine–gynae integrated; limited cardiometabolic pathways | Endocrine-focused with external referrals |
| **5. Quality Monitoring & Feedback Systems** | Patient satisfaction collected; no HCP evaluation | Patient satisfaction documented; limited HCP review | Patient satisfaction + HCP evaluations | HCP evaluations only; no patient satisfaction system | HCP evaluations documented; no formal satisfaction system | No patient satisfaction or HCP evaluations | HCP evaluations only | No satisfaction or HCP evaluations documented |

**Abbreviations:**
**EMR**, electronic medical record; **EPR**, electronic patient record; **HCP**, healthcare practitioner; **MDT**, multidisciplinary team; **NHS**, National Health Service; **PCOS**, polycystic ovary syndrome; **UK**, United Kingdom.
